# Prediction Model for Mild Cognitive Impairment in Older Chinese Patients With Cerebral Small Vessel Disease Based on XGBoost Algorithms and Shapley Additive Explanations

**DOI:** 10.1002/cns.71044

**Published:** 2026-07-29

**Authors:** Peng Gao, Jingjing Su, Xiaoming Ma, Xinwei Ma, Yujia Chen, Jiahui Shen, Yijie Wu, Cheng Shi, Jingwei Li

**Affiliations:** ^1^ Department of Clinical Psychology The Third Affiliated Hospital of Soochow University Changzhou China; ^2^ Department of Neurology The Second Affiliated Hospital of Nanjing Medical University Nanjing China; ^3^ Department of Neurology, Suzhou Hospital, Affiliated Hospital of Medical School Nanjing University Suzhou China; ^4^ Suzhou Key Laboratory of Integrated Stroke Prevention Treatment and Rehabilitation Suzhou China; ^5^ Department of Neurology, Drum Tower Hospital, Affiliated Hospital of Medical School Nanjing University Nanjing China; ^6^ Department of Radiology, Suzhou Hospital, Affiliated Hospital of Medical School Nanjing University Suzhou China; ^7^ Department of Neurology, Nanjing Drum Tower Hospital Clinical College of Traditional Chinese and Western Medicine Nanjing University of Chinese Medicine Nanjing China; ^8^ Suzhou Research Centre of Medical School Nanjing University Suzhou China; ^9^ Department of Cardiology Suzhou BenQ Medical Center Suzhou China; ^10^ Department of Neurology, Medical School and the State Key Laboratory of Pharmaceutical Biotechnology, Drum Tower Hospital Nanjing University Nanjing China; ^11^ Institute of Brain Sciences Nanjing University Nanjing China; ^12^ Jiangsu Key Laboratory for Molecular Medicine Medical School of Nanjing University Nanjing China; ^13^ Jiangsu Province Stroke Center for Diagnosis and Therapy Nanjing China; ^14^ Nanjing Neurology Clinic Medical Center Nanjing China

**Keywords:** CSVd, IGF‐1, MCI, prediction model, SHAP, XGBoost

## Abstract

**Objective:**

This study aims to evaluate cognitive function in patients with Cerebral Small Vessel Disease (CSVD) and investigate its association with variables such as serum Insulin‐like Growth Factor‐1 (IGF‐1). Artificial intelligence algorithms, specifically eXtreme Gradient Boosting (XGBoost) and SHapley Additive exPlanations (SHAP), were utilized for analysis and interpretation.

**Methods:**

A total of 216 patients diagnosed with CSVD were enrolled from the Department of Neurology, Third Affiliated Hospital of Soochow University, between November 2019 and August 2020. Clinical and biochemical data—including triglycerides, total cholesterol, low‐density lipoprotein cholesterol, fasting blood glucose, glycosylated hemoglobin (HbA1c), fasting insulin, C‐peptide, anti‐human insulin antibodies, and IGF‐1—were obtained under standardized laboratory protocols. Cognitive function was assessed using the Montreal Cognitive Assessment (MoCA). Based on cognitive performance, patients were categorized into CSVD with cognitive impairment and CSVD without cognitive impairment.

**Results:**

The original cohort included 216 patients with CSVD, comprising 50 patients with MCI and 166 patients without MCI. To address class imbalance during model development, SMOTE‐NC was applied within the development dataset. The XGBoost model achieved a precision of 0.790, recall of 0.901, F1 score of 0.820, accuracy of 0.833, and Cohen's kappa coefficient of 0.667 in the validation cohort. Feature importance analysis identified key predictors, while SHAP values enabled intuitive visualization of each feature's impact. Decision Curve Analysis (DCA) confirmed the model's clinical utility and net benefit, underscoring its potential for early MCI detection and targeted intervention to improve patient outcomes.

**Conclusion:**

Combining XGBoost and SHAP enhances model interpretability, facilitating the identification of critical risk factors such as reduced IGF‐1 levels in MoCA‐defined MCI in patients with CSVD. This AI‐driven approach offers a valuable tool for informing treatment decisions and optimizing healthcare resource allocation.

## Graphical abstract

An interpretable XGBoost‐SHAP model predicted MoCA‐defined cognitive impairment in older patients with cerebral small vessel disease and identified reduced IGF‐1 as the leading predictor. The model showed strong validation performance and clinical utility, supporting early risk stratification.

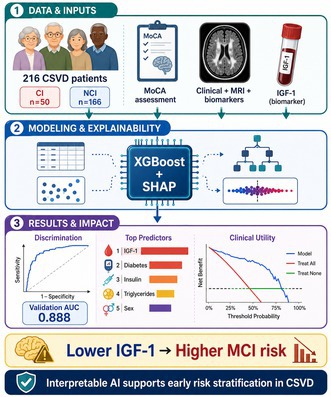

**Abbreviations**
AUC: Area Under the Curve
CSVD: cerebral small vessel disease
CT: computed tomography
DCA: Decision Curve Analysis
FLAIR: Fluid Attenuation Inversion Recovery
IGF‐1: insulin‐like growth factor‐1
IGF‐1: insulin‐like growth factor‐1
LI: Lacunar Infarction
MCI: Mild Cognitive Impairment
MMSE: Mini‐Mental State Examination
MoCA: The Montreal Cognitive Assessment
MRI: magnetic resonance imaging
PSCI: post‐stroke cognitive impairment
PSCID: post‐stroke cognitive impairment with dementia
ROC: Receiver Operating Characteristic Curve
WMH: White Matter Hyperintensity
WML: White Matter Lesion

## 1 Introduction

Cerebral Small Vessel Disease (CSVD) has emerged as a critical focus in neurological research. Despite evidence suggesting relatively low rates of disability and mortality [1, 2], CSVD is associated with a significantly elevated risk of stroke recurrence compared to large vessel disease. Defined as a syndrome encompassing clinical, cognitive, and pathological alterations, CSVD results from structural or functional impairment of cerebral small vessels—including small arteries, penetrating arterioles, venules, and capillaries [3]. Although some CSVD manifestations may be clinically silent or associated with relatively low immediate disability and mortality, CSVD is not benign. Accumulating small‐vessel injury is associated with stroke recurrence, cognitive impairment, gait disturbance, psychiatric symptoms, and urinary dysfunction. Among these consequences, cognitive decline is particularly burdensome because it may progressively impair independence and impose long‐term socioeconomic pressure on families and healthcare systems. Advancements in magnetic resonance imaging (MRI) and demographic shifts toward an aging population have contributed to increased incidence and detection rates. White matter hyperintensities and rarefaction, hallmark radiological features of CSVD, are prevalent in older adults—affecting approximately 80% of healthy individuals aged 60 and nearly all by age 90 [4].

MoCA‐defined MCI in patients with CSVD may involve multiple domains, including memory, visuospatial processing, attention, and executive function, and may progress from MCI to vascular cognitive impairment or dementia.

Previous studies have identified several risk factors contributing to post‐stroke cognitive impairment, including hyperglycemia, cerebral microvascular pathology, and severe hypoglycemia [5, 6]. With the growing prevalence of MoCA‐defined MCI in patients with CSVD, identifying simple, effective, and reliable biomarkers for early prediction has become imperative. Furthermore, machine learning techniques offer an opportunity to elucidate quantitative associations between biomarkers and clinical outcomes.

Insulin‐like Growth Factor‐1 (IGF‐1), a 7.5 kDa single‐chain peptide, is involved in a wide range of physiological and metabolic processes, including growth, development, aging, and lifespan regulation [7]. Under normal physiological conditions, IGF‐1 is extensively expressed in the central nervous system (CNS) [8]. Prior research has linked IGF‐1 deficiency with an increased risk of ischemic stroke, particularly in individuals with obesity or type 2 diabetes, suggesting its potential utility as a biomarker for ischemic risk stratification [9].

Emerging evidence supports the pivotal role of IGF‐1 in neurogenesis and its strong correlation with cognitive decline [8, 9, 10, 11]. IGF‐1 contributes to homeostatic regulation in hyperglycemia, improves insulin sensitivity, reduces amyloid‐beta (A*β*) protein–induced neurotoxicity, inhibits tau hyperphosphorylation, attenuates nitric oxide (NO)–mediated neurotoxicity, and mitigates cerebral ischemia and hypoxia—all of which collectively contribute to neuroprotection and reduced cognitive deterioration risk [11, 12, 13].

XGBoost [14] is a scalable implementation of gradient‐boosted decision trees that sequentially builds an ensemble of weak learners by optimizing a regularized objective function. Compared with conventional linear models, XGBoost can capture nonlinear associations and higher‐order interactions among heterogeneous clinical, biochemical, and imaging variables. This property is particularly relevant for MoCA‐defined MCI in patients with CSVD, in which vascular, metabolic, neuroendocrine, and neuroimaging factors may interact in complex ways. The model was trained and fine‐tuned using five‐fold cross‐validation to assess the association between serum IGF‐1 levels and cognitive function in patients with CSVD.

However, ensemble tree models are often criticized for limited transparency. Therefore, we applied SHapley Additive exPlanations [15, 16] (SHAP), a game‐theory‐based model interpretation framework, to quantify the contribution of each predictor to both individual‐level and global model predictions. SHAP values allow the direction and magnitude of each variable's contribution to predicted MCI risk to be visualized, thereby improving the interpretability of the XGBoost model.

## 2 Materials and Methods

### 2.1 Patients and File Documentation

Patients enrolled in this study were admitted to the Department of Neurology at the Third Affiliated Hospital of Soochow University between November 2019 and August 2020 [17]. Each patient underwent a comprehensive clinical evaluation and brain MRI, with diagnoses of CSVD confirmed independently by radiologists. The most prevalent neuroimaging findings included White Matter Hyperintensities (WMH) and Lacunar Infarctions (LI). WMH presented as hyperintense on T2‐weighted sequences and as isointense or mildly hypointense on T1‐weighted sequences—though not to the degree of cerebrospinal fluid. On computed tomography (CT), these lesions appeared as areas of white matter rarefaction or hypodensity. LI was defined as round or oval lesions, 3–15 mm in diameter, observable on T1‐ and T2‐weighted MRI, typically accompanied by a perilesional halo on Fluid‐Attenuated Inversion Recovery (FLAIR) sequences, often described as “capped” lesions [18]. DICOM images were reviewed independently by two neurologists, with discrepancies adjudicated by a senior specialist.

### 2.2 Inclusion and Exclusion Criteria

All participants were required to meet the following inclusion criteria:

1. Age between 50 and 85 years, physically capable, and able to comply with study procedures;
2. Diagnosis of CSVD based on the imaging criteria established by the 2013 International Vascular Cognitive and Behavioral Disorders Society and the 2015 Chinese Consensus on the Diagnosis and Treatment of Cerebral Small Vessel Disease. All participants underwent multimodal MRI, including conventional MRI, MRA, MRV, and DWI sequences.

Exclusion criteria were as follows:

1. Severe visual or auditory impairments, or motor disabilities impairing the ability to complete cognitive assessments:
2. History of significant endocrine disorders, including diabetic ketoacidosis coma, hyperthyroidism, hypothyroidism, systemic lupus erythematosus, among others;
3. Presence of conditions or prior CNS insults known to affect cognitive function, such as brain tumors, severe hepatic or renal dysfunction, traumatic brain injury, neurosyphilis, or epilepsy;
4. Pre‐existing diagnoses of other forms of dementia prior to CSVD, including AD, Lewy body dementia, or related neurodegenerative conditions.

### 2.3 IGF‐1 Testing Standard

At 8:00 AM on the morning following admission, 3 mL of fasting venous blood was collected from the antecubital vein of each participant. Samples were centrifuged at 4°C at 4000 rpm for 20 min, and the resulting serum was aliquoted and stored at −80°C until analysis. Serum IGF‐1 concentrations were measured using a standardized enzyme‐linked immunosorbent assay (ELISA) kit, with absorbance read at 450 nm. IGF‐1 levels (ng/ml) were calculated according to the manufacturer's protocol.

### 2.4 Diagnose of MoCA‐Defined MCI in Patients With CSVD

Cognitive function was evaluated using the Montreal Cognitive Assessment (MoCA) in a quiet, distraction‐free environment without visible clocks. Prior to the assessment, patients engaged in 5–10 min of informal conversation with the evaluator to establish rapport.

MoCA [19], recognized for its greater sensitivity compared to the Mini‐Mental State Examination (MMSE), serves as a more effective instrument for detecting cognitive impairment [20]. Its application facilitates the early identification of MCI, enabling timely intervention and potentially enhancing patients' quality of life. The assessment evaluates the following domains: (1) Visuospatial and executive function; (2) Naming; (3) Immediate and delayed recall; (4) Attention and calculation; (5) Language; (6) Abstraction; and (7) Temporal and spatial orientation.

The total score ranges from 0 to 30, with an additional point awarded to individuals with fewer than 12 years of education. A score ≥ 26 is considered within the normal cognitive range, while lower scores indicate cognitive decline.

One additional point was added for participants with fewer than 12 years of education. Participants with a pre‐existing clinical diagnosis of dementia before CSVD, including Alzheimer's disease, Lewy body dementia, or other neurodegenerative dementias, were excluded. No additional lower MoCA boundary was applied in the primary analysis; therefore, the outcome should be interpreted as MoCA‐defined MCI in patients with CSVD rather than a full etiological diagnosis of dementia.

### 2.5 Statistical Analysis

Patients were categorized into MCI and non‐MCI groups based on diagnostic outcomes, and variables were compared across groups. For instances of minor missing data, multiple imputation by chained equations (MICE) was conducted separately within each group to prevent feature leakage. The MCI group included 50 patients; the non‐MCI group comprised 166 patients. To address the class imbalance, the Synthetic Minority Over‐sampling Technique for Nominal and Continuous variables (SMOTE‐NC) [21] was employed, producing a balanced dataset.

To address class imbalance during model development, the Synthetic Minority Over‐sampling Technique for Nominal and Continuous variables (SMOTE‐NC) was applied only within the development dataset. The original cohort consisted of 216 patients, including 50 patients with MCI and 166 patients without MCI. XGBoost model hyperparameters were optimized using a predefined parameter grid, with fivefold cross‐validation implemented to mitigate overfitting. The optimized model was applied to the validation cohort, and performance was assessed using a confusion matrix.

### 2.6 XGBoost Model Development and SHAP Interpretation

XGBoost was selected as the primary prediction algorithm because it is well suited for tabular clinical data and can model nonlinear relationships and interactions among predictors. Candidate predictors included demographic characteristics, vascular risk factors, biochemical markers, and neuroimaging variables. Hyperparameters were optimized using grid search with fivefold cross‐validation in the development dataset. The final optimized model [22] was then evaluated in the validation cohort using discrimination and classification metrics.

To improve model transparency, SHAP analysis was performed after model training. SHAP assigns each feature a Shapley value, representing its contribution to an individual prediction relative to the average model output. Global SHAP summary plots were used to rank predictors according to their overall contribution, while the distribution of SHAP values was used to examine whether higher or lower values of each predictor increased the predicted probability of MCI.

Thus, XGBoost and SHAP were compared according to their analytical roles: XGBoost was responsible for model construction and prediction, whereas SHAP was responsible for interpreting the trained XGBoost model by estimating feature‐specific contributions to the predicted probability of MCI.

## 3 Results

Following the establishment of the baseline model, a grid search optimization was conducted to iteratively enhance model performance. The optimal hyperparameter configuration identified was:_rounds = 200, max_depth = 5, eta = 0.25, gamma = 0.01, colsample_bytree = 0.75, and min_child_weight = 1.

### 3.1 Demographic Information and Characteristics of Participants

See Table 1.

**TABLE 1:** Demographic and cognitive characteristics of the study population after applying the SMOTE‐NC technique (*n* = 332).

Characteristic	CSVD‐related MCI (*n* = 166)	NCI (*n* = 166)	All (*n* = 332)	*p*
Age (years)	69.30 ± 8.38	64.03 ± 7.36	68.57 ± 8.73	< 0.001
Education Years	8.87 ± 2.49	9.38 ± 1.56	9.12 ± 2.09	0.025
HbA1c (mmol/L)	6.86 ± 1.76	5.96 ± 0.85	6.41 ± 1.45	< 0.001
Systolic blood pressure (mmHg)	148.01 ± 20.59	144.05 ± 17.38	146.03 ± 19.13	0.060
Diastolic blood pressure (mmHg)	85.54 ± 11.68	84.57 ± 9.02	85.05 ± 10.43	0.400
Insulin (pmol/L)	62.80 ± 41.54	47.82 ± 24.38	55.31 ± 34.82	< 0.001
C‐Peptide (pmol/L)	873.16 ± 396.05	731.66 ± 217.29	802.41 ± 326.72	< 0.001
Anti‐Insulin Antibodies (IU/ml)	4.72 ± 2.77	3.83 ± 1.65	4.28 ± 2.32	< 0.001
IGF‐1 (ng/ml)	114.11 ± 28.99	147.67 ± 38.61	130.89 ± 38.00	< 0.001
FBG (mmol/L)	6.52 ± 2.49	5.58 ± 1.55	6.05 ± 2.12	< 0.001
Total cholesterol (mmol/L)	4.51 ± 1.20	4.34 ± 1.00	4.43 ± 1.11	0.152
Triglyceride (mmol/L)	1.79 ± 1.13	1.54 ± 0.86	1.66 ± 1.01	0.026
Low‐density lipoprotein (mmol/L)	2.68 ± 1.02	2.66 ± 0.76	2.67 ± 0.90	0.859
MoCA Score	21.68 ± 1.92	26.87 ± 1.06	24.06 ± 3.04	< 0.001
Sex (%)				
Male	115 (69.28)	105 (63.25)	220 (66.27)	0.296
Female	51 (30.72)	61 (36.75)	112 (33.73)	
History of Diabetes mellitus (%)				
Yes	102 (61.45)	23 (13.86)	125 (37.65)	< 0.001
No	64 (38.55)	143 (86.14)	207 (62.35)	
White matter lesions (%)				
Yes	46 (27.71)	19 (11.45)	65 (19.58)	< 0.001
No	120 (72.29)	147 (88.55)	267 (80.42)	

Abbreviations: FBG, fasting blood glucose; MCI, mild cognitive impairment; NCI, non‐cognitive impairment.

### 3.2 Model Performance Assessment

**FIGURE 1:**
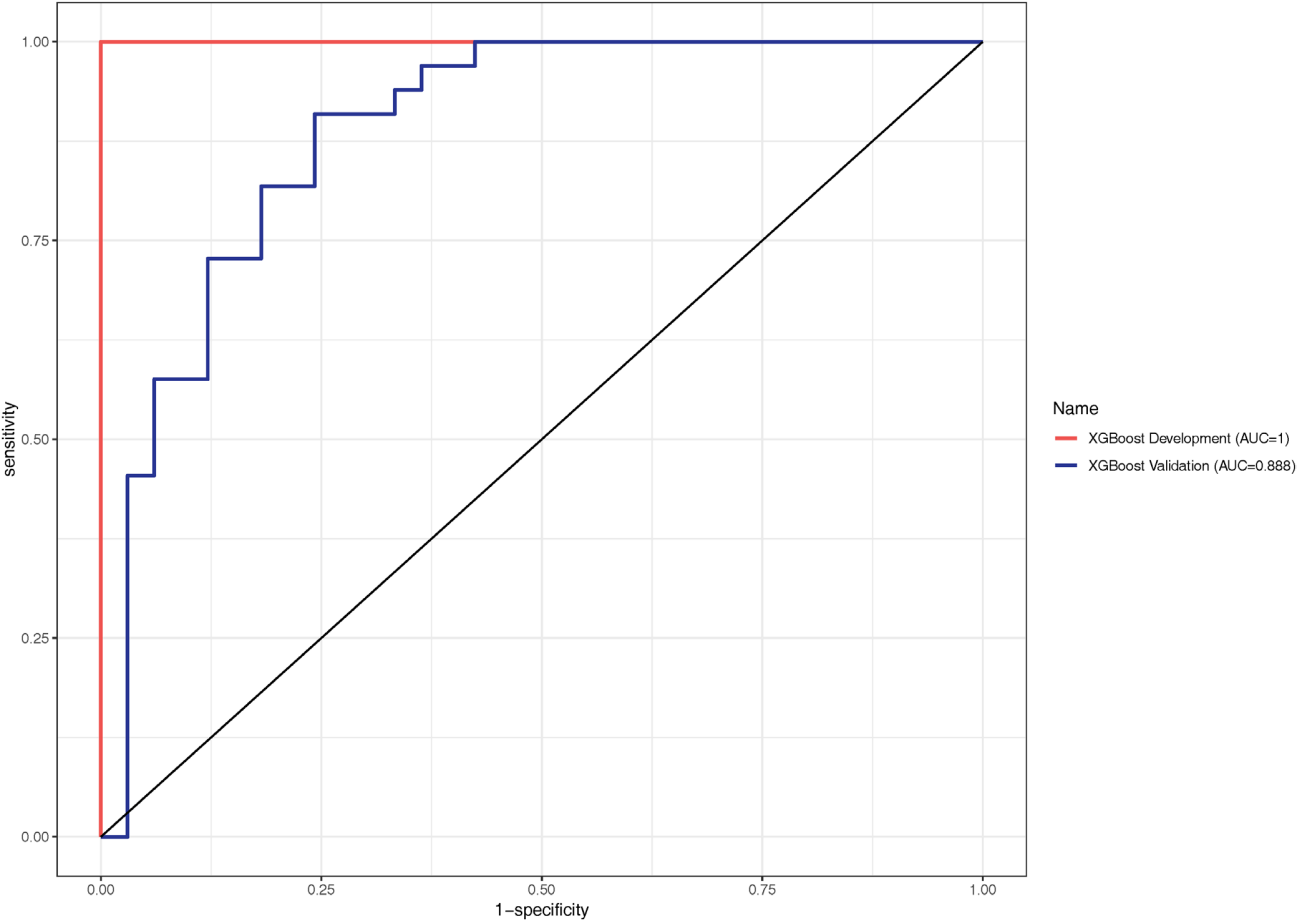
ROC curve for the development and validation cohorts. AUC, area under the curve; ROC, receiver operating characteristic curve.

Receiver operating characteristic (ROC) curves were plotted for both groups to evaluate model discrimination performance (Figure 1).

To comprehensively assess model performance, a confusion matrix was constructed using the validation cohort (Table 2), enabling the calculation of precision, recall, accuracy, F1 score, Cohen's kappa, sensitivity, specificity, positive predictive value (PPV), and negative predictive value (NPV). This multifaceted evaluation provided a robust measure of the model's effectiveness in predicting MCI.

**TABLE 2:** Confusion matrix from XGBoost.

XGBoost		Prediction			Precision	0.790
		MCI	Non‐MCI	Sensitivity	Recall	0.901
Reference	MCI	30	3	90.91%	F1 score	0.820
	Non‐MCI	8	25	75.76%	Accuracy	0.833
	PPV	78.95%	89.29%		Kappa	0.667

*Note:* Blue for True samples, while Green for Positive/Negative Precision Value.

**FIGURE 2:**
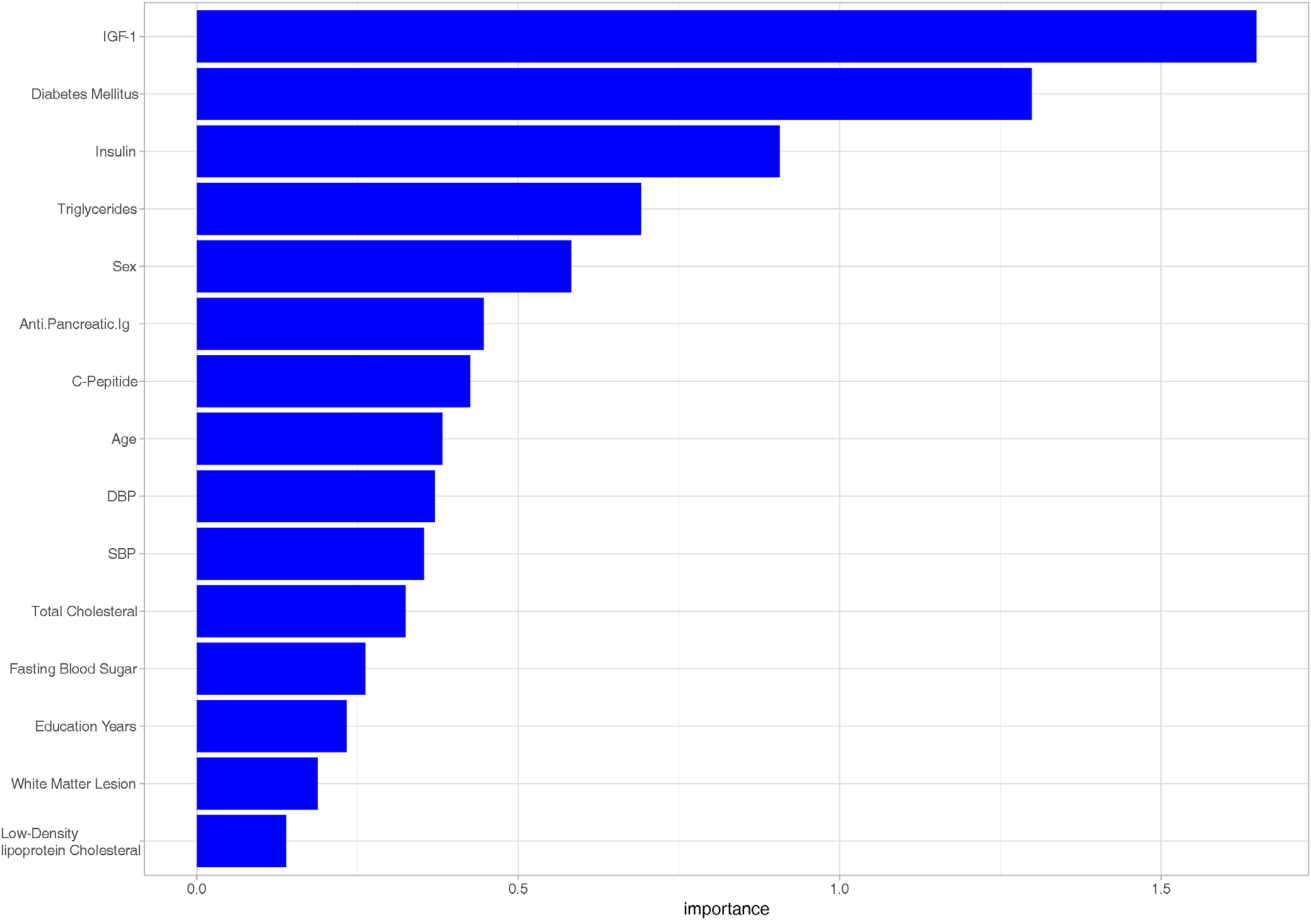
Feature importance derived from the XGBoost model.

Feature importance was extracted from the XGBoost model to quantify the relative contribution of each variable to the predictive outcomes (Figure 2). This analysis offered valuable insights into the most influential predictors, enhancing interpretability and revealing the key factors driving model performance.

**FIGURE 3:**
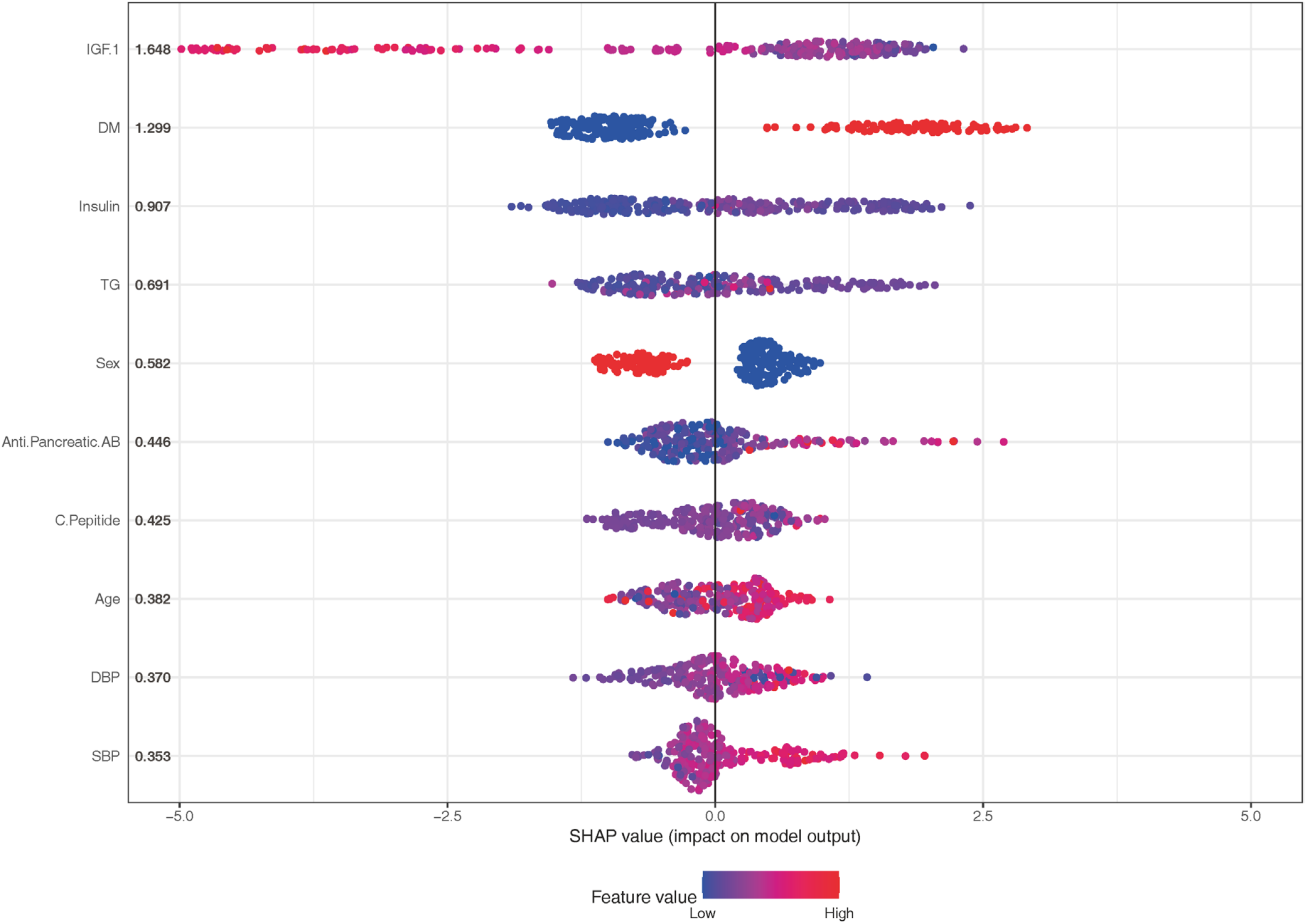
SHAP summary plot of the XGBoost model.

To further elucidate the model's internal decision‐making processes, SHAP was applied (Figure 3). By normalizing the influence of measurement units, SHAP values enabled a more intuitive and comprehensive visualization of each feature's impact on the prediction, thereby improving transparency and interpretability.

**FIGURE 4:**
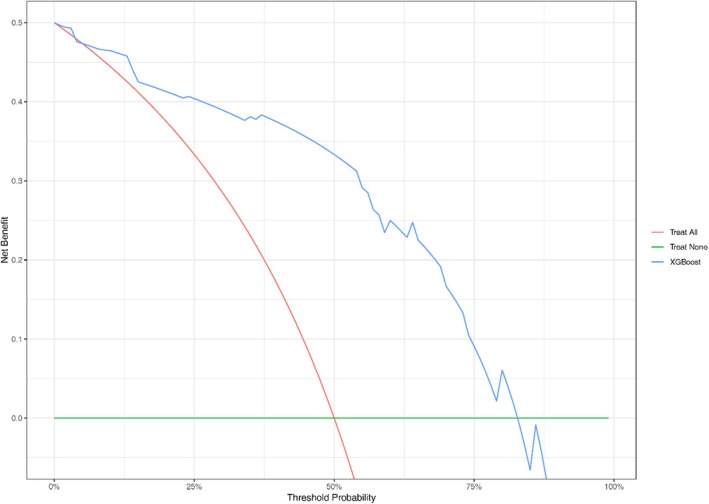
Decision curve analysis of the XGBoost model.

In addition, a decision curve analysis (DCA) was conducted using the validation cohort to evaluate the clinical utility and net benefit of the model (Figure 4). This approach assessed the model's potential to improve clinical decision‐making by estimating the range of threshold probabilities at which the model would provide a meaningful advantage in patient care.

## 4 Discussion

The main added value of the present analytic framework lies not merely in the use of an “AI” algorithm, but in the combination of nonlinear prediction and model interpretability. Conventional group‐level analyses can identify variables that differ between MCI and non‐MCI groups, but they provide limited information about individualized risk profiles or complex interactions among predictors. In contrast, XGBoost can integrate demographic, metabolic, biochemical, and imaging variables within a nonlinear prediction framework. SHAP further decomposes the model output into feature‐specific contributions, allowing both global ranking of predictors and patient‐level explanation of predicted MCI risk. Therefore, the XGBoost‐SHAP framework provides a clinically interpretable risk stratification tool rather than a simple association analysis. Importantly, this framework is not intended to replace conventional clinical assessment or causal inference. Rather, it may serve as an auxiliary risk‐stratification approach by integrating routinely available clinical and biochemical variables and presenting the contribution of each predictor in an interpretable manner.

Diabetes and insulin‐resistance‐related markers were also identified as relevant predictors in the model. This finding is biologically plausible because diabetes is associated with metabolic dysregulation, microvascular injury, and multidomain cognitive dysfunction. However, the present study was designed to develop an interpretable prediction model rather than to establish causal mechanisms. Therefore, these associations should be interpreted as hypothesis‐generating and require confirmation in future longitudinal and mechanistic studies.

Baseline patient characteristics are detailed in Table 1. The proposed model integrates the XGBoost algorithm, with its predictive performance illustrated via ROC curves (Figure 1) and a confusion matrix (Table 2), both indicating strong model performance. Feature importance outputs from the XGBoost model (Figure 2), complemented by SHAP analysis (Figure 3), further demonstrate the model's interpretability and predictive accuracy. Figure 4 illustrates the DCA results, indicating that across a threshold probability range of 10%–85%, the model consistently outperformed both full intervention and no intervention strategies in decision‐making efficacy.

Given the rising incidence of CSVD‐associated cognitive decline, identifying simple and effective biomarkers is essential. A comprehensive approach combining qualitative and quantitative analyses—particularly through advanced machine learning algorithms—holds promise for improving early detection and management of MCI.

In this study, IGF‐1 demonstrated superior predictive performance. As a 7.5 kDa single‐chain peptide implicated in diverse physiological and metabolic processes, IGF‐1 plays a pivotal role in growth, development, lifespan regulation, and aging [7]. Under normal physiological conditions, it is broadly expressed in the CNS. Prior studies have established a correlation between IGF‐1 levels and ischemic stroke risk, with reduced circulating IGF‐1 representing a notable risk factor in obese and diabetic populations. Serum IGF‐1 may serve as a critical biomarker for assessing ischemic disease risk, particularly in individuals with type 2 diabetes or central obesity [9]. Accumulating evidence highlights its essential role in neurogenesis and its involvement in cognitive dysfunction. IGF‐1 contributes to homeostatic regulation in hyperglycemic states, ameliorates insulin resistance, and attenuates A*β*‐induced neurotoxicity, tau hyperphosphorylation, and NO‐mediated neuronal damage, thereby exerting neuroprotective effects. These mechanisms collectively support improved cerebral perfusion and oxygenation, ultimately mitigating cognitive decline [12, 13, 23]. This study examined the association between serum IGF‐1 levels, related influencing factors, and cognitive performance in patients with CSVD, aiming to provide a theoretical foundation for therapeutic strategies.

Additionally, patients with CSVD and comorbid diabetes exhibited lower MoCA scores relative to non‐diabetic counterparts, suggesting that diabetes may aggravate CSVD‐related MCI in this population. The impact was particularly pronounced in domains including visuospatial and executive function, language, memory, and orientation. This observation aligns with prior research indicating that individuals with MCI and type 2 diabetes experience multidomain cognitive deficits encompassing attention, language, and executive abilities. Hyperglycemia has also been linked to an elevated risk of dementia [24].

As illustrated in Figure 3, patients with CSVD and comorbid diabetes exhibited significantly lower MoCA scores than those without diabetes, indicating that diabetes may accelerate the progression of CSVD‐related MCI in individuals with CSVD. This decline was particularly evident in visuospatial and executive function, language, memory, and orientation. These findings are consistent with previous studies demonstrating that individuals with both MCI and type 2 diabetes often experience multidomain cognitive deficits, encompassing attention, language, and executive function. Chronic hyperglycemia has been strongly associated with an increased risk of developing dementia. Despite growing evidence, the mechanisms underlying CSVD‐related MCI in diabetic individuals remain only partially understood. Proposed pathophysiological mechanisms include insulin resistance, oxidative stress, pancreatic *β*‐cell dysfunction, neuronal apoptosis, and mitochondrial impairment. The present findings revealed that CSVD individuals with related MCI exhibited elevated serum insulin and anti‐insulin antibody levels compared to cognitively intact individuals, suggesting a potential link between insulin resistance and diabetes‐related cognitive decline. Insulin resistance is known to accelerate biological aging and brain atrophy by promoting the formation of advanced glycation end products (AGEs) and reactive oxygen species (ROS), thereby impairing cognitive function [25].

Increased insulin resistance also enhances the activity of glycogen synthase kinase‐3 beta (GSK‐3*β*) [26], which facilitates tau protein hyperphosphorylation and A*β* accumulation, inhibits acetylcholine synthesis [27, 28], and induces neuronal apoptosis—all of which contribute to cognitive deterioration. Another hypothesized mechanism involves the insulin‐degrading enzyme (IDE) [27], which degrades A*β* and prevents the formation of senile plaques. Elevated insulin concentrations in the brain may compete with A*β* for IDE binding, reducing A*β* clearance and accelerating neurodegenerative processes. Reduced IDE levels have been observed in the brains of patients with dementia, particularly within the hippocampus. Furthermore, individuals with MCI and diabetes exhibit significantly lower serum IDE levels compared to cognitively normal controls [29, 30]. IDE levels show a positive correlation with MoCA scores, supporting the notion that IDE activity and insulin resistance are key contributors to the pathogenesis of diabetes‐associated cognitive impairment [31, 32].

IGF‐1, a polypeptide neurotrophic factor, plays an essential role in neuronal development and is closely associated with higher‐order cognitive functions, including memory. Prior research has demonstrated a significant correlation between IGF‐1 levels and insulin resistance, wherein reduced IGF‐1 concentrations coincide with diminished insulin sensitivity, contributing to the development of insulin resistance [33]. In models of age‐related insulin resistance and IGF‐1 deficiency, central administration of IGF‐1 has been shown to restore systemic insulin responsiveness, improve glycemic control, and mitigate insulin resistance, thereby lowering the risk of cognitive decline in diabetic populations. Nevertheless, the pathophysiological mechanisms underlying diabetes‐related cognitive impairment remain incompletely defined. Contributing factors may include insulin resistance, oxidative stress [34], *β*‐cell dysfunction, neuronal apoptosis, and mitochondrial impairment. The current study observed elevated serum insulin and anti‐insulin antibody levels in patients with CSVD exhibiting related MCI relative to those without, implicating insulin resistance as a potential driver of neurodegenerative processes in diabetes. Insulin resistance may accelerate brain aging and atrophy by promoting the accumulation of AGEs and ROS, both of which detrimentally affect cognitive performance.

A key innovation of this study lies in the application of artificial intelligence (AI) techniques to manage and interpret high‐dimensional datasets, enabling a robust analysis of the association between serum IGF‐1 levels and MCI in patients with CSVD. Moreover, the study identified additional risk factors for CSVD‐related MCI, including a history of diabetes, female sex, and the presence of anti‐insulin antibodies.

Despite these contributions, certain limitations warrant consideration. The single‐center design may introduce selection and diagnostic bias, particularly due to inter‐observer variability among radiologists. Furthermore, the precise mechanisms linking diabetes and related MCI within the context of CSVD remain to be fully elucidated. Notwithstanding these limitations, the findings offer valuable insights into the interplay between IGF‐1, insulin resistance, and cognitive dysfunction, potentially informing the development of targeted therapeutic interventions and guiding more efficient allocation of clinical resources.

The observed decline in serum IGF‐1 levels and its association with cognitive deterioration in patients with CSVD may be attributable to the neuroprotective properties of IGF‐1. Preclinical research has demonstrated that IGF‐1 confers substantial neuroprotection in models of cerebral ischemia [35]. Beyond promoting the development and survival of embryonic neurons, IGF‐1 supports the functional integrity of mature neurons and facilitates the repair and regeneration of damaged neural tissue. Importantly, its neuroprotective efficacy remains consistent across various delivery routes, including intracerebroventricular, intravenous, and intranasal administration.

This study presents several notable strengths. The integration of AI techniques enabled the effective analysis of high‐dimensional datasets and uncovered a strong association between serum IGF‐1 levels and MCI in patients with CSVD. Furthermore, additional risk factors contributing to cognitive decline were identified, including a history of diabetes, female sex, and the presence of anti‐insulin antibodies.

Nonetheless, certain limitations should be acknowledged. As a single‐center investigation, the study may be subject to selection and diagnostic bias, particularly from radiological assessments. Moreover, the mechanistic pathways linking diabetes to related MCI in CSVD patients require further elucidation through longitudinal and mechanistic studies. Despite these constraints, the findings provide meaningful insights into the role of IGF‐1 and associated risk factors in CSVD‐related cognitive impairment, with implications for the development of targeted therapeutic approaches and the optimized distribution of healthcare resources.

## 5 Conclusion

The application of XGBoost combined with SHAP interpretation notably enhances the interpretability of the machine learning model, addressing the “black‐box” nature typically associated with AI‐based predictions. This approach allows for transparent identification of critical risk factors—particularly reduced IGF‐1 levels—associated with MCI in patients with CSVD. Such an AI‐driven framework holds promise for guiding clinical decision‐making, enabling more individualized treatment planning, and improving resource allocation within the healthcare system.

## Author Contributions

Peng Gao, Jingwei Li, and Jingjing Su conceptualized the study and oversaw its overall execution. Xiaoming Ma, Peng Gao, and Jingjing Su were involved in data analysis, manuscript drafting, and revisions; these authors contributed equal to this work. Xinwei Ma and Peng Gao were responsible for data acquisition, verification, and preliminary data cleaning.

## Funding

This study was supported by the Jiangsu Commission of Health (K2023023, LKZ2024006), the Suzhou Municipal Health Commission (DZXYJ202313), and the Nanjing Drum Tower Hospital Clinical Research Project (2021‐LCYJ‐MS‐21).

## Ethics Statement

All participants voluntarily submitted relevant medical history data, underwent cognitive assessments, and contributed laboratory data retrieved from the hospital's standardized Health Information System (HIS). Ethical approval for the study was granted by the Ethics Committee of the Third Affiliated Hospital of Soochow University (2023‐S‐001).

## Consent

The requirement for informed consent from the study patients was waived due to the retrospective study design.

## Conflicts of Interest

The authors declare no conflicts of interest.

## Data Availability

The data that support the findings of this study are available from the corresponding author upon reasonable request.
